# Breast Milk Iodine Concentration Is Associated with Infant Growth, Independent of Maternal Weight

**DOI:** 10.3390/nu12020358

**Published:** 2020-01-30

**Authors:** Lindsay Ellsworth, Harlan McCaffery, Emma Harman, Jillian Abbott, Brigid Gregg

**Affiliations:** 1 Division of Neonatal-Perinatal Medicine, Department of Pediatrics, University of Michigan, Ann Arbor, MI 48109, USA; ellsworl@med.umich.edu; 2 Center for Human Growth and Development, University of Michigan, Ann Arbor, MI 48109, USA; hmccaff@umich.edu; 3 School of Public Health, University of Michigan, Ann Arbor, MI 48109, USA; erharman@umich.edu; 4 Metals Laboratory, Division of Clinical Biochemistry and Immunology, Mayo Clinic, Rochester, MN 55905, USA; abbott.jillian@mayo.edu; 5 Division of Pediatric Endocrinology, Department of Pediatrics, University of Michigan, Ann Arbor, MI 48109, USA

**Keywords:** iodine status, human milk, lactation, infant growth

## Abstract

In breastfed infants, human milk provides the primary source of iodine to meet demands during this vulnerable period of growth and development. Iodine is a key micronutrient that plays an essential role in hormone synthesis. Despite the importance of iodine, there is limited understanding of the maternal factors that influence milk iodine content and how milk iodine intake during infancy is related to postnatal growth. We examined breast milk samples from near 2 weeks and 2 months post-partum in a mother-infant dyad cohort of mothers with pre-pregnancy weight status defined by body mass index (BMI). Normal (NW, BMI < 25.0 kg/m^2^) is compared to overweight/obesity (OW/OB, BMI ≥ 25.0 kg/m^2^). The milk iodine concentration was determined by inductively coupled plasma mass spectrometry. We evaluated the associations between iodine content at 2 weeks and infant anthropometrics over the first year of life using multivariable linear mixed modeling. Iodine concentrations generally decreased from 2 weeks to 2 months. We observed no significant difference in iodine based on maternal weight. A higher iodine concentration at 2 weeks was associated with a larger increase in infant weight-for-age and weight-for-length Z-score change per month from 2 weeks to 1 year. This pilot study shows that early iodine intake may influence infant growth trajectory independent of maternal pre-pregnancy weight status.

## 1. Introduction

Iodine is a key micronutrient required for adequate growth and development through its critical role in thyroid hormone synthesis [1]. Deficiency in iodine during the neonatal and infant periods may result in impairments in brain development, cognitive outcomes, motor function, and growth stunting. Exposure to excess iodine can lead to iodine-induced hyperthyroidism or hypothyroidism [2,3,4,5]. Newborns are a particularly vulnerable population for iodine related disorders given their low storage of iodine in the thyroid at birth and their relatively high requirements for iodine relative to their body size [2,3,6,7,8,9].

Exclusively breast-fed infants depend primarily on breast milk iodine to meet their daily iodine needs [7,10]. The United States Institute of Medicine recommends 110 µg/day iodine intake for infants from birth to 6 months of age, while the World Health Organization (WHO) recommends 90 µg/day to achieve adequate intake [11,12]. In order to support fetal and neonatal development, the American Thyroid Association recommends all pregnant and lactating women supplement their iodine intake with 150 µg/day iodine [3,13]. Iodine supplementation for mothers is intended to achieve the United States Institute of Medicine recommendation of 290 µg/day iodine intake and WHO recommendation of 250 µg/day. Through this degree of supplementation during lactation, the goal is to reach a sufficient breast milk iodine concentration (BMIC) to support a breast-fed infant’s daily requirements [5,13]. However, the exact BMIC needed to meet infant needs is not universally defined [3,14].

BMIC is impacted by the maternal degree of iodine sufficiency from dietary iodine sources or supplementation. BMIC also varies based on the time point during lactation, maternal smoking, maternal health conditions, and with day to day fluctuation [3,15,16,17,18,19]. In establishing maternal iodine insufficiency, iodine is thought to be secreted into breast milk rather than excreted into the urine, to promote adequate levels of intake for offspring. The concentrating ability of mammary epithelial cells has been modeled with in vivo rat studies showing that the sodium/iodine symporter (NIS) mediates transport; however, published studies on the activity of the NIS in human mammary epithelial cells remain limited [20]. BMIC is a stronger marker of maternal iodine status than urine concentration [7,10,14,21,22,23,24,25]. BMIC is the greatest in the colostrum and then decreases in early lactation, with an over 40% reduction over the first 6 months of lactation [2,3,6,7,10,15,16]. To date, the impact of maternal health factors on BMIC has not been well described.

In pregnant and non-pregnant women, body mass index (BMI) has been negatively associated with urinary iodine concentration, which is measured as an indicator of iodine status [26,27,28]. Iodine deficiency has also been reported to be higher in those with morbid obesity [29]. The evaluation of BMIC in the establishment of obesity during pregnancy and lactation has not been well studied. Given the documented relationship with BMI, BMIC may have an important connection with rising levels of obesity in adulthood, as well as a potential contributing factor to childhood obesity. Iodine studies in mother-infant cohorts have shown mixed results regarding birth anthropometric outcomes, with some studies associated with an increased mean birth weight with improved maternal iodine status [30,31,32,33,34]. Postnatal studies of iodine levels have shown an impact on growth factors but have not shown a consistent association with infant growth [1,31,35,36,37,38].

With this study, we aimed to determine maternal BMIC over the first 2 months of lactation in mothers with normal weight compared to mothers with elevated pre-pregnancy BMI to characterize the impact of maternal metabolic factors on BMIC. We also assessed the impact of BMIC on infant growth trajectory over the first year of life while taking into account maternal pre-pregnancy weight status to gain a greater understanding of the impact of iodine on post-natal growth.

## 2. Materials and Methods

### 2.1. Subjects and Methods

This pilot study included a subset of 57 mother-infant dyads recruited in 2016 to 2018 at the University of Michigan and St. Joseph Mercy Ann Arbor Hospital during hospital admission at the time of infant delivery as part of the prospective Infant Metabolism and Gestational Endocrinopathies (IMAGE) cohort. This study was developed to detect differences in breast milk insulin concentration. This is a secondary analysis of the collected breast milk samples. The mothers included in this study were enrolled if they (1) planned to breastfeed their infant, (2) were > 18 years of age, and (3) had a healthy singleton infant delivered at ≥ 35 weeks gestation. Maternal health conditions, including obesity, gestational diabetes, and polycystic ovary syndrome were included. Maternal demographics and health history were obtained through an electronic medical record review and paper surveys. The maternal pre-pregnancy weight status was determined through a calculation of BMI using obstetrics recorded pre-pregnancy or the early first trimester weight and height as documented in the medical record. Mother-infant dyads were categorized by maternal pre-pregnancy weight status as normal weight (NW, BMI < 25.0 kg/m^2^) or overweight/obese (OW/OB, BMI ≥ 25.0 kg/m^2^).

The study was approved by the institutional review boards at the University of Michigan (HUM00107801, approved 01/05/2016) and St. Joseph Mercy Ann Arbor Hospital (HSR-17–1686, approved 03/07/17). Mothers gave written informed consent for themselves and assent for their infants prior to participation in the study. The study was conducted in accordance with the protocol approved by the institutional review boards. Participants received reimbursement for their involvement in this study.

### 2.2. Milk Collection

A written milk collection protocol was provided to the mothers, and in person verbal instruction was given by the study team. Mothers were instructed to collect a 25 mL milk sample on the morning of their infant’s 2 week (transitional milk) and 2 month (mature milk) routine well-child visit. The milk collection protocol was based on the published methods by Fields and Demerath, with modifications as described [39]. Mothers collected milk samples between 8:00 and 10:00 am and at least 2 h after feeding their infant by hand expression or pumping of an entire breast based on the maternal preference of expression type. Mothers expressed milk into a large container and then mixed the milk by inversion before the milk was transferred to five glass vials (5 mL each). Samples were immediately placed in the mother’s home freezer and then transported on ice to the clinic for storage at −20 °C prior to transport on ice to the final storage location at −80 °C within one week. The milk was thawed on ice for subsequent analyses, at which time 250 µL was aliquoted for further iodine assessment after refreezing at −80 °C and shipment on dry ice.

### 2.3. Laboratory Analysis

Frozen whole milk 250 µL aliquots were shipped on dry ice to the Mayo Clinic Metals Laboratory (Rochester, MN) for iodine analysis. PerkinElmer Sciex ELAN Dynamic Reaction Cell (DRC) II Inductively Coupled Plasma Mass Spectrometer (ICP-MS), manufactured in Waltham, Massachusetts, USA was used for testing. The gold standard of ICP-MS was used [3]. All samples had one freeze-thaw cycle additional before dilution and were tested within an International Standards Organization (ISO) class 7 cleanroom. The analysis performed followed the standard operating procedure of a test that was developed and consistent with Clinical Laboratory Improvement Amendments (CLIA) requirements. The method used commercially prepared calibrators (Inorganic Ventures, Christiansburg, VA, USA) in 1% TMAH, 7.5 g/L NaCl and 0.5 g/L CaCl_2_ matrix with iodine concentrations at 0, 10, 100, 500, 1000, 5000 µg/L, having an analytical measurement range (AMR) from 10 to 40,000 µg/L, with the use of 10,000 µg/L and 40,000 µg/L linearity standards. Results above linearity were diluted with SRW (≥18 MΩ cm Special Reagent Water using a NANOpure system, Thermo Scientific, Waltham, MA, USA).

To assess the degree of error for the test method, a recovery study was performed on 3 breast milk samples by spiking them with iodine calibration standards (0, 100 and 500 µg/L) and two levels of Quality Control UTAK Serum Trace Element (UTAK Laboratories, Inc. Valencia, CA, USA), as shown in Table 1. Initial dilutions were repeated five times in duplicate for an intra-assay coefficient of variation (CV). Samples were then run with subsequent loads over a period of five days for an inter-assay CV. The average percentages of recovery were all within 10% of the expected values and <10% for the intra and inter-assay CV.

The same levels of UTAK Serum Trace Elements were used for the Quality Control Analysis; levels were analyzed identically to those of the patient samples in duplicate. QC was run after every 20 samples and at the end of each load. During the sample preparation step each specimen was thoroughly vortexed immediately before performing a two-step dilution using a Hamilton MicorLab diluter (Hamilton Company, Reno, NV, USA) to aspirate 60 µg/L of the sample and 60 µg/L of the Iodine 0 Solution, combined with Iodine Diluent (50 µg/L Te (30% HCl *v*/*v*) and 10 µg/L Rh (21% HCl *v*/*v*) in 1% TMAH) for a total of 3 mL. The iodine concentration was measured in ng/mL. Samples were analyzed in duplicate, and the results were averaged for reporting.

One milk sample at 2 weeks had a significantly elevated iodine concentration upon initial analysis, which was consistent on repeat analysis at a separate run time point; given this outlier concentration of >7000 ng/mL without biological plausibility, this value was truncated to the next highest iodine concentration value in our cohort (649.1 ng/mL). A potential etiology may include iodine exposure peri-partum through antiseptics; however, this was unable to be verified.

### 2.4. Infant Anthropometric Measurements

Infant health history was reviewed from the electronic medical record, including documentation of birth history during hospital admission and follow-up routine outpatient well-child pediatric visits. The type of nutritional intake, such as human milk, formula, or combinations, was determined from the pediatrician notes at the well-child visits. Infant growth anthropometric measures were extracted from the medical record measurements for weight and length obtained in the hospital at birth and during outpatient well-child visits at 2 weeks, 2 months, 6 months, and 1 year. Age and sex specific WHO Z-scores for weight-for-age (WFA), length-for-age (LFA), and weight-for-length (WFL) were extracted from the medical record [40].

### 2.5. Statistical Analysis

The statistical analysis was completed using R version 3.6.1 (R Foundation for Statistical Computing, Vienna, Austria) and the package nlme version 3.1–140 for this pilot study. Descriptive statistics, comparisons of overweight and obese mothers, and unadjusted comparisons of log(BMIC) were done using Fisher’s exact tests or *t*-tests, as appropriate, on all dyads with complete data for each test (*n* = 49 for comparisons involving 2 week BMIC; *n* = 50–57 for all other covariates).

Linear mixed models with random intercepts at the subject level were fit using the 35 dyads that had exclusively breast milk feedings at 2 weeks and 2 months, with BMIC measured at 2 weeks. WFA Z-score (WFAZ), LFA Z-score (LFAZ), and WFL Z-score (WFLZ) were considered dependent variables. Estimates were obtained for the fixed effects for the following independent variables: 2 weeks BMIC, time (in months, 0.5, 2, 6, 12), birth anthropometric Z-score, maternal BMI, gender, interaction of maternal BMI and time, and interaction of 2 week BMIC and time. The interaction of BMIC and time was used to test the association between BMIC and infant growth, and the interaction of maternal BMI and time was included to control for the effects of maternal BMI on infant growth. An initial time point of 2 weeks was used due to the anticipated fluid shift in the first 2 weeks of life in newborns. Parameter estimates were considered statistically significant at *p* < 0.05.

## 3. Results

This mother-infant cohort included 57 dyads with infant growth measures from birth to 1 year of age, with the descriptive analysis shown in Table 2. Of the 57 dyads, 25 mothers provided milk samples only at 2 weeks, 8 mothers provided milk samples only at 2 months, and 24 mothers provided milk samples at both 2 weeks and 2 months. Mothers in our Midwestern population were primarily Caucasian in the OW/OB maternal group compared to the NW maternal group. Infants were delivered at term gestational age, with 47% male infants. At the 2 week time point of milk collection (mean 16.5 days, SD 3.0 days), 49 milk samples were available for analysis. At the 2 month time point of time collection (mean 63.0 days, SD 5.6 days), 32 milk samples were available for analysis. At the 2 week time point with milk samples collected, 86% of the infants were reported to be exclusively breastfed, 10% received breast milk and formula, 4% did not have their nutrition intake reported, and no infants were exclusively formula fed. By the 2 month time point after the milk samples were collected, 81% of infants received exclusively breast milk, 12% of infants received breast milk and formula, 6% of infants did not have their nutritional intake documented, and no infants were exclusively formula fed. Higher rates of formula supplementation were seen among mothers with OW/OB at 2 weeks (17%) and 2 months (20%). The exact amount of formula supplementation was not quantified.

**Figure 1:**
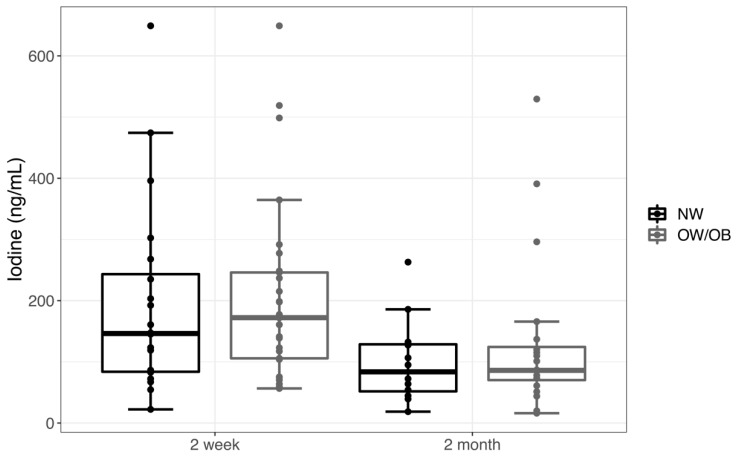
Breast milk iodine concentration (based on the maternal pre-pregnancy weight status for normal weight (NW) compared to overweight and obese (OW/OB) mothers) in transitional milk at 2 weeks (NW *n* = 20, OW/OB *n* = 29) and mature milk at 2 months (NW *n* = 12, OW/OB *n* = 20). Data are represented as a box plot, with the box showing median (IQR) and the whiskers equal to the farthest observation less than 1.5 times IQR from the box edge.

BMIC ranged from 16.0 to 649.1 ng/mL in samples collected near 2 weeks and 2 months. The median BMIC at 2 weeks was 160.7 ng/mL, and, by 2 months, it was 86.0 ng/mL. For comparisons of the mean BMIC, we used a log-transformation BMIC to satisfy the normality assumptions (*p* = 0.73 and 0.56 for the 2 week and 2 month BMIC, respectively, with a Shapiro-Wilk test). There was no significant difference in the log-BMIC between NW and OW/OB mothers at 2 weeks (*p* = 0.54) or at 2 months (*p* = 0.57). The log-BMIC decreased from 2 weeks to 2 months by 0.59 (39%, *p* = 0.007) in the 24 mothers who had iodine measurements at both time points. There was no impact of infant sex, maternal county of residence, or maternal income on BMIC. Untransformed BMIC is shown by the weight status and time of measurement in Figure 1.

**Figure 2:**
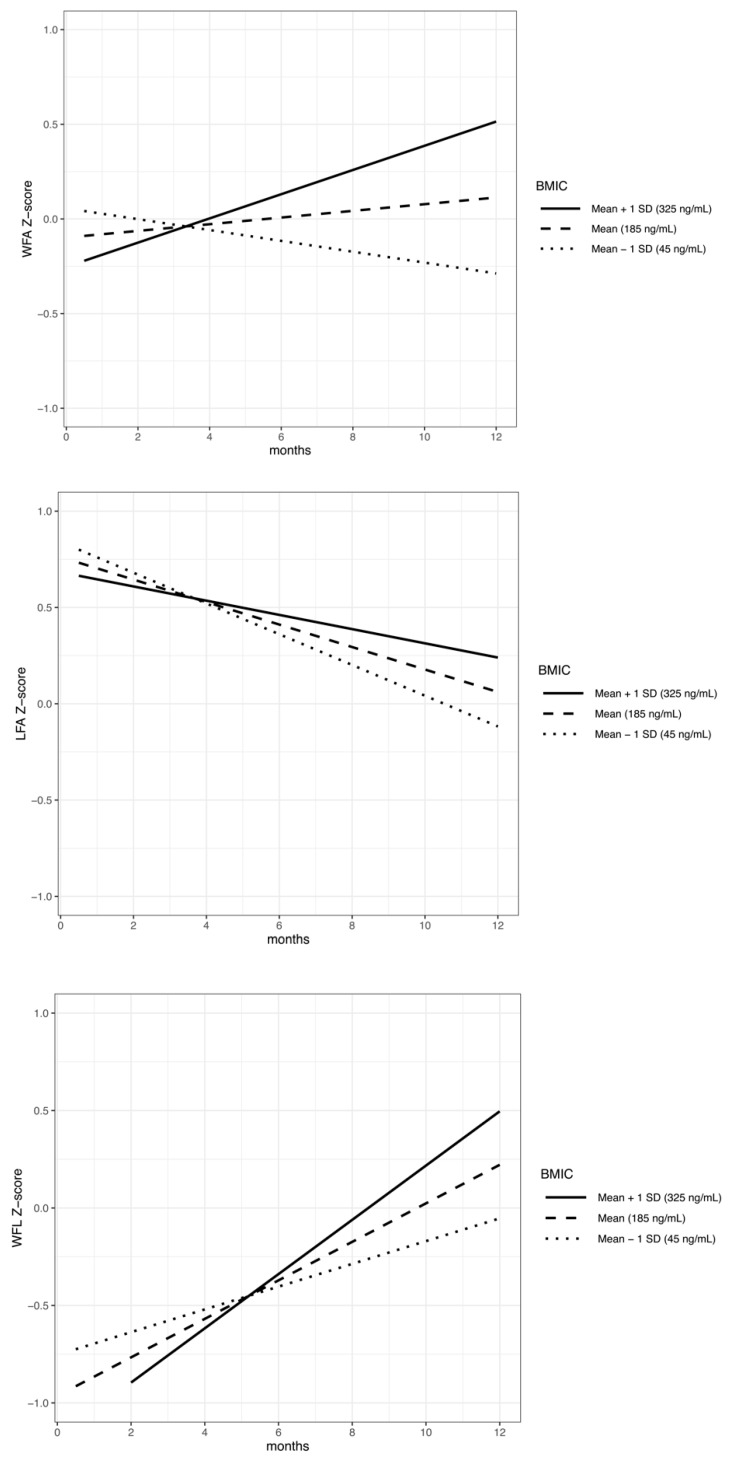
Infant growth anthropometric Z-score from 2 weeks to 1 year, with BMIC predicted by linear mixed models (model *n* = 35). Graphs depict the difference in the predicted growth lines for the mean ± 1 standard deviation BMIC as measured at 2 weeks for dyads with a male infant, the mean birth anthropometric Z-score, and the mean maternal BMI. Interaction effect of BMIC and time (months) on infant WFA (β = 0.00033, *p* < 0.001), infant LFA (β = 0.00015, *p* = 0.154), and infant WFL (β = 0.00029, *p* = 0.021). Abbreviations: weight-for-age Z-score (WFA), length-for-age Z-score (LFA), and weight-for-length Z-score (WFL); breast milk iodine concentration (BMIC).

We then explored the impact of maternal weight and BMIC on infant growth from 2 weeks to 1 year using linear mixed models, with a subset of dyads who had complete data for all variables included in the model, and who were exclusively breastfed at 2 weeks and 2 months (*n* = 35). We evaluated infant growth by testing the interactions of maternal BMI with time, and BMIC at 2 weeks with time, using an anthropometric Z-score through the first year of life, with the WFA, LFA, and WFL Z-scores as dependent variables. We found no significant interactions between maternal pre-pregnancy BMI and time for WFA (β = −0.00126, *p* = 0.534), LFA (β = 0.00359, *p* = 0.118), or WFL (β = −0.00452, *p* = 0.089) Z-score changes per month from 2 weeks to 1 year. A higher milk iodine concentration at 2 weeks was associated with a larger increase in infant WFA (β = 0.00033, *p* = 0.0007) and WFL Z-score (β = 0.00029, *p* = 0.0212) change per month from 2 weeks to 1 year, with no significant association with LFA (β = 0.00015, *p* = 0.154), as shown in Table 3. Figure 2 represents the model interaction terms of BMIC and time, showing the predicted WFAZ, LFAZ, and WFLZ for a male infant with a mean birth Z-score, breastfed by a mother with mean BMI based on a 2 week BMIC mean, mean +1 standard deviation (SD), and −1 SD.

## 4. Discussion

In the maternal-infant dyads in this study, transitional milk BMIC was positively associated with infant WFA and WFL Z-score changes over the first year of life, and these iodine-related growth differences were not related to maternal pre-pregnancy weight status based on this pilot study. The importance of early milk iodine may point to a critical period of offspring development during high vulnerability and a time during which formula fed infants may consume less iodine than breastfed infants.

Milk iodine concentrations were not associated with maternal pre-pregnancy weight status in our cohort. While literature on adult obesity has described iodine deficiency as associated with elevated BMI, we did not find similar findings related to BMI as a marker of iodine status [26,27,28,29]. Limited literature on BMIC during lactation has focused on the maternal health influences on milk iodine concentration [26,27,29]. A study by Dumrongwongsiri, in Thailand, showed an association of BMIC with maternal weight; however, there was no association with maternal age or lactation stage [41]. This study did not define the time point of maternal weight measurement and may not reflect the weight prior to pregnancy, as assessed in our study. It is important for future studies on BMIC to include an analysis of maternal pre-pregnancy weight status in order to understand the role of maternal health on milk micronutrient composition.

The milk iodine concentrations in our cohort showed considerable variability. The BMIC to achieve adequate iodine delivery to infants is debated, with the lower most BMIC proposed to range from 60 to 150 ng/mL [3,13,14,22]. While exact values of the ideal BMIC for sufficient infant intake ranges are not well described, a goal of 150 ng/mL in breast milk has been reported [3]. The levels in our study showed a high degree of values below 150 ng/mL at 2 weeks (47%) and even more below 150 ng/mL at 2 months (81%). These results are higher than those of a study from the Netherlands, showing that 33% of infants were anticipated to be iodine deficient based on BMIC <1.1 µmol/L (139 ng/mL) [42]. Mothers with low BMIC may signify a population with iodine insufficiency, as BMIC has been shown to be positively correlated with maternal urinary iodine concentration, the standard measure of iodine sufficiency [43]. Currently, the United States has salt iodization programs, with a reported use near 90% due to a decline in iodized salt into the 1990s, based on National Health and Nutrition Examination Survey (NHANES) data [44]. Additionally, the NHANES reports on iodine status during pregnancy showed a concerning degree of iodine insufficiency (56.9%) in 2005–2008 [45]. Previous cohorts from Boston have shown a similar degree of iodine insufficiency (47%) based on estimated infant milk intake; however, this group had higher milk iodine concentrations (median 155 ng/mL) at a median of 48 days collection compared to BMIC at our 2 month time point (median 86 ng/mL) [43]. A meta-analysis by Nazeri described overall lower mature milk iodine level in iodine sufficient countries (mean 71.5 ng/mL) [46]. This difference may represent population, geographic, or nutritional intake differences.

While our cohort may be a population at risk for infant iodine insufficiency based on BMIC, we also noted some elevated BMIC levels in both NW and OW/OB throughout the first 2 months of lactation. The level of BMIC that may lead to iodine excess has not been defined in the same manner that urinary iodine concentration >300 ng/mL in children and >500 ng/mL in pregnancy is defined as iodine excess [47]. Prior studies of high iodine exposure through elevated water iodine levels by Liu identified a BMIC >200 ng/mL in mothers with high iodine intake [48]. A further evaluation of excessive iodine exposure through breast milk is necessary to determine the safe levels of exposure, particularly in mothers at risk for iodine excess, including those with high dietary, oral, or topical iodine exposure [49].

Our data on BMIC overall, independent of maternal pre-pregnancy weight status, suggests a relationship between infant iodine exposure and growth, with a higher BMIC at 2 weeks associated with an increased change in Z-score for WFA and WFL over the first year of life. However, the clinical implications of these small growth changes are unclear, and future long-term studies into childhood growth and adiposity are needed. Prior literature has focused on the impact of iodine status and supplementation on prenatal growth, with a systematic review showing low quality evidence for increased birth weight in infants of mothers with severe iodine deficiency who are receiving iodine supplementation [31]. Postnatal growth studies are limited in both observational and iodine supplementation intervention studies during lactation [31]. In a study performed in China by Yang, a maternal iodine deficiency based on urinary iodine levels <50 µg/L during lactation was linked to a decreased infant WFA and LFA for infants <6 months of age, with positive effects of maternal iodine status on infant weight; however, BMIC was not measured to determine infant intake [1]. A review of survey data by Mason showed a positive relationship with iodine salt use and increased infant WFA and mid-upper-arm-circumference at 2 years. This study also assessed maternal BMI, showing that the positive relationship between iodinated salt use and WFA was increasingly greater with a lower maternal BMI [50]. However, there have been opposing trials and systematic reviews showing no impact of iodine intake on infant growth. In a multicenter randomized control trial of iodine supplementation during infancy in breastfeeding infants compared to placebo or formula fed infants, there was no difference found for 12 month WFA Z-score or WFL Z-score in infants receiving high or low iodine supplementation; however, infants receiving no intervention had higher LFA Z-scores and formula fed infants had higher WFA, WFL, and LFA Z-scores [51]. In a systematic review by Farebrother, on the impact of iodine supplementation on postnatal growth outcomes based on limited supplementation studies during pregnancy and one randomized clinical trial of preterm infant formula supplementation, there was no difference at 12 and 24 months in infant weight, length, or head circumference with maternal iodine supplementation [31].

Our prospective observational cohort study without specific iodine supplementation evaluated the association of BMIC with infant anthropometrics and found positive associations between growth over the first year and early BMIC near 2 weeks. The potential mechanisms for iodine related infant growth may be related to the role of iodine in the endocrine pathways of the thyroid hormone (TH) and growth hormone-insulin-like growth factor (GH-IGF) axis [31,32]. Zimmerman showed in a prospective intervention of iodine repletion in school age children that there was improved somatic growth in the WFA and head circumference-for-age Z-score associated with increased IGF-I and IGF binding protein-3 (IGFBP-3) concentrations in children [38]. Future work evaluating the link between BMIC, infant somatic growth, and growth related hormone levels (TSH, free thyroxine, IGF-1, and IGFBP-3) may provide further insight into mediators and identify areas for intervention for the promotion of iodine sufficient status during lactation.

This study provides insight into longitudinal BMIC in a United States Midwestern population, with corresponding assessments of infant growth through the first year of life in this pilot study. The strength of this study is that the population was selected for their initial plans to breastfeed without selection for predetermined maternal iodine status. This pilot study is limited by the small sample size used for its secondary analysis, which the initial study was not powered for; the mixed modeling involved a large number of parameters for the sample size, which could raise questions about the generalizability of these models among the larger population. However, the significant associations with BMIC and infant growth seen with these small numbers highlights the importance of consideration for BMIC analysis in more expansive mother-infant lactation focused cohorts to further understand this potential early programming influence. Additional limitations, including the assessment of infant growth outcomes, were measured by medical staff at pediatrician well-child visits, possibly resulting in inaccurate anthropometrics. Infant growth is complex, and this study is not able to address the numerous factors contributing to infant growth, which are not limited to genetic, environmental, later formula supplementation quantity, complimentary feedings, milk volume intake, and the complex composition of human milk, including macronutrients, micronutrients, bioactive factors, growth hormones, and immune factors [52,53]. Our cohort did not assess iodine status during pregnancy, which could impact fetal growth and development. Maternal iodine intake was also not quantified in this study. This will be important in future studies to provide a further explanation for the high BMIC among the population of lactating mothers. An assessment of infant iodine status will also be an important addition to future work to determine the relationship with BMIC based on detailed 24 h infant breast milk volume intake.

## 5. Conclusions

While the importance of maternal health during pregnancy and lactation in promoting infant growth and development cannot be understated, this study did not show the impact of maternal pre-pregnancy BMI on BMIC. We have identified a positive association between transitional BMIC and infant weight and weight-for-length growth over the first year of life based on a Z-score irrespective of maternal pre-pregnancy weight status. These findings highlight the critical need for knowledge of breast milk composition, particularly in micronutrients (such as iodine), which play a key role in infant growth. A further evaluation of the complex components in breast milk and the impact of these components during ongoing organ development in infancy is necessary to promote targeted interventions to support optimal childhood health.

## Figures and Tables

**Table 1:** Performance characteristics of the iodine assay.

Breast Milk Precision and Recovery					
Target	0 Standard	100 µg/L	500 µg/L	NR UTAK QC	HR UTAK QC
Mean	42.89	145.56 µg/L	551.52 µg/L	49 ng/mL	186 ng/mL
CV%	9.32%	6.65%	0.43%	9.6%	2.5%
Recovery		97.40 µg/L	450.9 µg/L	46 µg/L	179 µg/L
% Recovery		97.40%	90.18%	93.88%	96.24%

**Table 2:** Participant demographics.

Participant Characteristics	NW	OW/OB	*p*
	(*n* = 24)	(*n* = 33)	
Maternal Age: years, mean (SD)	31.00 (3.66)	31.61 (3.06)	0.5
* Maternal Pre-Pregnancy BMI: kg/m^2^, mean (SD)	21.25 (1.99)	30.95 (4.69)	<0.001
* Maternal Race/Ethnicity			0.002
Caucasian, no. (%)	11 (46)	28 (85) *	
African American, no. (%)	2 (8)	1 (3)	
Hispanic/Latino, no. (%)	4 (17)	1 (3)	
Asian/Pacific Islander, no. (%)	6 (25)	1 (3)	
Indian, no. (%)	1 (4)	0 (0)	
N/A, no. (%)	0 (0)	2 (6)	
Gestational Age: weeks, mean (SD)	39.5 (1.0)	39.1 (1.3)	0.16
Mode of Delivery			0.39
Vaginal, no. (%)	18 (75)	20 (60)	
C-section, no. (%)	6 (25)	13 (40)	
Country			0.1
Washtenaw, no. (%)	18 (75)	17 (52)	
Other, no. (%)	6 (25)	16 (49)	
Maternal income: dollars, mean (SD)			0.04
<60,000, no. (%)	21 (88)	19 (68)	
>60,000, no. (%)	3 (12)	9 (32)	
Unknown, no. (%)	0 (0)	5 (15)	
Smoker			>0.99
Yes, no. (%)	1 (4)	1 (3)	
No, no. (%)	19 (80)	27 (82)	
N/A, no. (%)	4 (17)	5 (15)	
Infant birth weight: kg mean (SD)	3.42 (0.36)	3.52 (0.42)	0.35
Infant birth weight: Z-score mean (SD)	0.33 (0.78)	0.42 (0.81)	0.68
Infant sex			>0.99
Male, no. (%)	11 (54)	16 (49)	
Female, no. (%)	13 (54)	17 (51)	
Infant age at 2 week time point: days, mean (SD)	15.9 (3)	16.9 (3)	0.27
Infant age at 2 month time point: days, mean (SD)	62.0 (7.2)	62.5 (6.7)	0.8

Descriptive statistics on maternal and infant demographics from the cohort of 57 mother-infant dyads presented as the mean (standard deviation) or number (percentage). The sample size is slightly smaller than the totals presented for infants aged at 2 weeks (missing data for 9 infants). Statistical analysis using a *t*-test or Fischer’s exact test. * represents statistical significance, with a *p*-value < 0.05. Abbreviations: normal weight (NW), overweight and obesity (OW/OB), not available (N/A) number (no.). BMI *p* < 0.001, Maternal race/ethnicity *p* = 0.002.

**Table 3:** Association of maternal weight status and 2 week breast milk iodine concentration with change in weight-for-age, length-for-age, and weight-for-length Z-score across the first year of life.

Variables		WFAZ	LFAZ	WFLZ
	**Model *n***	**Difference (β (*p*-value)) in change in infant anthropometry Z-score**		
**BMIC at 2 weeks (ng/mL)**	35	−0.1883 (0.706)	1.23581 (0.028) *	−1.1945 (0.053)
**Time (months)**	35	−0.0011 (0.170)	−0.00056 (0.527)	−0.0015 (0.136)
**Birth WFAZ**	35	0.53027 (0.0006) *	−0.18117 (0.002) *	0.16434 (0.016) *
**Maternal BMI (kg/m^2^)**	35	−0.00532 (0.769)	0.42163 (0.0004) *	0.24395 (0.022) *
**Infant sex**	35	0.23087 (0.247)	−0.03543 (0.080)	0.01866 (0.411)
**Maternal BMI interaction with time**	35	−0.00126 (0.536)	0.00359 (0.118)	−0.00452 (0.089)
**BMIC at 2 weeks interaction with time**	35	0.00033 (0.0007) *	0.00015 (0.154)	0.00029 (0.021) *

* Statistical significance based on an estimation of the fixed effects in linear mixed models, with a significance threshold at *p* < 0.05. Abbreviations: weight-for-age Z-score (WFAZ), length-for-age Z-score (LFAZ), weight-for-length Z-score (WFLZ), body mass index (BMI), and breastmilk iodine concentration (BMIC).

## References

[1] Yang J., Zhu L., Li X., Zheng H., Wang Z., Hao Z., Liu Y. (2017). Maternal iodine status during lactation and infant weight and length in Henan Province, China. BMC Pregnancy Childbirth.

[2] Azizi F., Smyth P. (2009). Breastfeeding and maternal and infant iodine nutrition. Clin. Endocrinol. (Oxf).

[3] Dror D.K., Allen L.H. (2018). Iodine in Human Milk: A Systematic Review. Adv. Nutr. (Bethesda, Md.).

[4] Michaelsen K.F., Larsen P.S., Thomsen B.L., Samuelson G. (1994). The Copenhagen Cohort Study on Infant Nutrition and Growth: Breast-milk intake, human milk macronutrient content, and influencing factors. Am. J. Clin. Nutr..

[5] Zimmermann M.B. (2011). The role of iodine in human growth and development. Semin. Cell Dev. Biol..

[6] Etling N., Padovani E., Fouque F., Tato L. (1986). First-month variations in total iodine content of human breast milks. Early Hum. Dev..

[7] Semba R.D., Delange F. (2001). Iodine in human milk: Perspectives for infant health. Nutr. Rev..

[8] van den Hove M.F., Beckers C., Devlieger H., de Zegher F., De Nayer P. (1999). Hormone synthesis and storage in the thyroid of human preterm and term newborns: Effect of thyroxine treatment. Biochimie.

[9] Zimmermann M.B. (2012). Are weaning infants at risk of iodine deficiency even in countries with established iodized salt programs?. Nestle Nutr. Inst. Workshop Ser..

[10] Mulrine H.M., Skeaff S.A., Ferguson E.L., Gray A.R., Valeix P. (2010). Breast-milk iodine concentration declines over the first 6 mo postpartum in iodine-deficient women. Am. J. Clin. Nutr..

[11] WHO (2007). Reaching Optimal Iodine Nutrition in Pregnant and Lactating Women and Young Children.

[12] Institute of Medicine (US). Panel on Micronutrients (2001). Dietary Reference Intakes for Vitamin A, Vitamin K, Arsenic, Boron, Chromium, Copper, Iodine, Iron, Manganese, Molybdenum, Nickel, Silicon, Vanadium, and Zinc. https://www.ncbi.nlm.nih.gov/books/NBK222310/.

[13] (2013). ACOG Committee opinion No. 549: Obesity in pregnancy. Obstet. Gynecol..

[14] Dold S., Zimmermann M.B., Baumgartner J., Davaz T., Galetti V., Braegger C., Andersson M. (2016). A dose-response crossover iodine balance study to determine iodine requirements in early infancy. Am. J. Clin. Nutr..

[15] Chierici R., Saccomandi D., Vigi V. (1999). Dietary supplements for the lactating mother: Influence on the trace element content of milk. Acta Paediatr. (Oslo, Norway: 1992) Suppl..

[16] Henjum S., Lilleengen A.M., Aakre I., Dudareva A., Gjengedal E.L.F., Meltzer H.M., Brantsaeter A.L. (2017). Suboptimal Iodine Concentration in Breastmilk and Inadequate Iodine Intake among Lactating Women in Norway. Nutrients.

[17] Osei J., Andersson M., Reijden O.V., Dold S., Smuts C.M., Baumgartner J. (2016). Breast-Milk Iodine Concentrations, Iodine Status, and Thyroid Function of Breastfed Infants Aged 2–4 Months and Their Mothers Residing in a South African Township. J. Clin. Res. Pediatr. Endocrinol..

[18] Sabatier M., Garcia-Rodenas C.L., Castro C.A., Kastenmayer P., Vigo M., Dubascoux S., Andrey D., Nicolas M., Payot J.R., Bordier V. (2019). Longitudinal Changes of Mineral Concentrations in Preterm and Term Human Milk from Lactating Swiss Women. Nutrients.

[19] Chen Y., Gao M., Bai Y., Hao Y., Chen W., Cui T., Guo W., Pan Z., Lin L., Wang C. (2019). Variation of iodine concentration in breast milk and urine in exclusively breastfeeding women and their infants during the first 24 wk after childbirth. Nutrition (Burbank, Los Angeles County, Calif.).

[20] Tazebay U.H., Wapnir I.L., Levy O., Dohan O., Zuckier L.S., Zhao Q.H., Deng H.F., Amenta P.S., Fineberg S., Pestell R.G. (2000). The mammary gland iodide transporter is expressed during lactation and in breast cancer. Nat. Med..

[21] Brown-Grant K. (1957). The iodide concentrating mechanism of the mammary gland. J. Physiol.

[22] Dold S., Zimmermann M.B., Aboussad A., Cherkaoui M., Jia Q., Jukic T., Kusic Z., Quirino A., Sang Z., San Luis T.O. (2017). Breast Milk Iodine Concentration Is a More Accurate Biomarker of Iodine Status Than Urinary Iodine Concentration in Exclusively Breastfeeding Women. J. Nutr..

[23] Eskin B.A., Bartuska D.G., Dunn M.R., Jacob G., Dratman M.B. (1967). Mammary gland dysplasia in iodine deficiency. Studies in rats. JAMA.

[24] Nazeri P., Dalili H., Mehrabi Y., Hedayati M., Mirmiran P., Azizi F. (2018). Breast Milk Iodine Concentration Rather than Maternal Urinary Iodine Is a Reliable Indicator for Monitoring Iodine Status of Breastfed Neonates. Biol. Trace Elem. Res..

[25] Strum J.M. (1979). Effect of iodide-deficiency on rat mammary gland. Virchows Arch. B Cell Pathol. Incl. Mol. Pathol..

[26] Robinson S.M., Crozier S.R., Miles E.A., Gale C.R., Calder P.C., Cooper C., Inskip H.M., Godfrey K.M. (2018). Preconception Maternal Iodine Status Is Positively Associated with IQ but Not with Measures of Executive Function in Childhood. J. Nutr..

[27] Soriguer F., Valdes S., Morcillo S., Esteva I., Almaraz M.C., de Adana M.S., Tapia M.J., Dominguez M., Gutierrez-Repiso C., Rubio-Martin E. (2011). Thyroid hormone levels predict the change in body weight: A prospective study. Eur. J. Clin. Invest..

[28] Torlinska B., Bath S.C., Janjua A., Boelaert K., Chan S.Y. (2018). Iodine Status during Pregnancy in a Region of Mild-to-Moderate Iodine Deficiency is not Associated with Adverse Obstetric Outcomes; Results from the Avon Longitudinal Study of Parents and Children (ALSPAC). Nutrients.

[29] Lecube A., Zafon C., Gromaz A., Fort J.M., Caubet E., Baena J.A., Tortosa F. (2015). Iodine deficiency is higher in morbid obesity in comparison with late after bariatric surgery and non-obese women. Obes. Surg..

[30] Alvarez-Pedrerol M., Guxens M., Mendez M., Canet Y., Martorell R., Espada M., Plana E., Rebagliato M., Sunyer J. (2009). Iodine levels and thyroid hormones in healthy pregnant women and birth weight of their offspring. Eur. J. Endocrinol..

[31] Farebrother J., Naude C.E., Nicol L., Sang Z., Yang Z., Jooste P.L., Andersson M., Zimmermann M.B. (2018). Effects of Iodized Salt and Iodine Supplements on Prenatal and Postnatal Growth: A Systematic Review. Adv. Nutr. (Bethesda, Md.).

[32] Gunnarsdottir I., Dahl L. (2012). Iodine intake in human nutrition: A systematic literature review. Food Nutr. Res..

[33] Rydbeck F., Rahman A., Grander M., Ekstrom E.C., Vahter M., Kippler M. (2014). Maternal urinary iodine concentration up to 1.0 mg/L is positively associated with birth weight, length, and head circumference of male offspring. J. Nutr..

[34] (2019). Iodine supplementation for women during the preconception, pregnancy and postpartum period - Harding, KB - 2017. Cochrane Libr..

[35] Aboud F.E., Bougma K., Lemma T., Marquis G.S. (2017). Evaluation of the effects of iodized salt on the mental development of preschool-aged children: A cluster randomized trial in northern Ethiopia. Matern. Child Nutr..

[36] Gowachirapant S., Jaiswal N., Melse-Boonstra A., Galetti V., Stinca S., Mackenzie I., Thomas S., Thomas T., Winichagoon P., Srinivasan K. (2017). Effect of iodine supplementation in pregnant women on child neurodevelopment: A randomised, double-blind, placebo-controlled trial. Lancet Diabetes Endocrinol..

[37] Zimmermann M.B., Connolly K., Bozo M., Bridson J., Rohner F., Grimci L. (2006). Iodine supplementation improves cognition in iodine-deficient schoolchildren in Albania: A randomized, controlled, double-blind study. Am. J. Clin. Nutr..

[38] Zimmermann M.B., Jooste P.L., Mabapa N.S., Schoeman S., Biebinger R., Mushaphi L.F., Mbhenyane X. (2007). Vitamin A supplementation in iodine-deficient African children decreases thyrotropin stimulation of the thyroid and reduces the goiter rate. Am. J. Clin. Nutr..

[39] Fields D.A., Demerath E.W. (2012). Relationship of insulin, glucose, leptin, IL-6 and TNF-alpha in human breast milk with infant growth and body composition. Pediatr. Obes..

[40] (2006). WHO Child Growth Standards based on length/height, weight and age. Acta Paediatr. (Oslo, Norway: 1992) Suppl..

[41] Dumrongwongsiri O., Chatvutinun S., Phoonlabdacha P., Sangcakul A., Chailurkit L.O., Siripinyanond A., Suthutvoravut U., Chongviriyaphan N. (2018). High Urinary Iodine Concentration Among Breastfed Infants and the Factors Associated with Iodine Content in Breast Milk. Biol. Trace Elem. Res..

[42] Stoutjesdijk E., Schaafsma A., Dijck-Brouwer D.A.J., Muskiet F.A.J. (2018). Iodine status during pregnancy and lactation: A pilot study in the Netherlands. Neth J. Med..

[43] Pearce E.N., Leung A.M., Blount B.C., Bazrafshan H.R., He X., Pino S., Valentin-Blasini L., Braverman L.E. (2007). Breast milk iodine and perchlorate concentrations in lactating Boston-area women. J. Clin. Endocrinol. Metab..

[44] Hollowell J.G., Staehling N.W., Hannon W.H., Flanders D.W., Gunter E.W., Maberly G.F., Braverman L.E., Pino S., Miller D.T., Garbe P.L. (1998). Iodine nutrition in the United States. Trends and public health implications: Iodine excretion data from National Health and Nutrition Examination Surveys I and III (1971–1974 and 1988–1994). J. Clin. Endocrinol. Metab..

[45] Caldwell K.L., Makhmudov A., Ely E., Jones R.L., Wang R.Y. (2011). Iodine status of the U.S. population, National Health and Nutrition Examination Survey, 2005–2006 and 2007–2008. Thyroid.

[46] Nazeri P., Kabir A., Dalili H., Mirmiran P., Azizi F. (2018). Breast-Milk Iodine Concentrations and Iodine Levels of Infants According to the Iodine Status of the Country of Residence: A Systematic Review and Meta-Analysis. Thyroid.

[47] WHO Assessment of Iodine Deficiency Disorders and Monitoring Their Elimination. https://apps.who.int/iris/bitstream/handle/10665/43781/9789241595827_eng.pdf.

[48] Liu L., Liu J., Wang D., Shen H., Jia Q. (2019). Effect of Urinary Iodine Concentration in Pregnant and Lactating Women, and in Their Infants Residing in Areas with Excessive Iodine in Drinking Water in Shanxi Province, China. Biol. Trace Elem. Res..

[49] (2006). Iodine. Drugs and Lactation Database (LactMed) [Internet].

[50] Mason J.B., Deitchler M., Gilman A., Gillenwater K., Shuaib M., Hotchkiss D., Mason K., Mock N., Sethuraman K. (2002). Iodine fortification is related to increased weight-for-age and birthweight in children in Asia. Food Nutr. Bull..

[51] Nazeri P., Tahmasebinejad Z., Mehrabi Y., Hedayati M., Mirmiran P., Azizi F. (2018). Lactating Mothers and Infants Residing in an Area with an Effective Salt Iodization Program Have No Need for Iodine Supplements: Results from a Double-Blind, Placebo-Controlled, Randomized Controlled Trial. Thyroid.

[52] Ballard O., Morrow A.L. (2013). Human milk composition: Nutrients and bioactive factors. Pediatr. Clin. N. Am..

[53] Eriksen K.G., Christensen S.H., Lind M.V., Michaelsen K.F. (2018). Human milk composition and infant growth. Curr. Opin. Clin. Nutr. Metab. Care.

